# Editorial: Immune dysfunction in acute pancreatitis: from bench to bedside research

**DOI:** 10.3389/fimmu.2024.1462563

**Published:** 2024-07-19

**Authors:** Wandong Hong, Maddalena Zippi, Gang Wang, Xi Jin, Wenhua He, Hemant Goyal

**Affiliations:** ^1^ Department of Gastroenterology and Hepatology, the First Affiliated Hospital of Wenzhou Medical University, Wenzhou, Zhejiang, China; ^2^ Unit of Gastroenterology and Digestive Endoscopy, Sandro Pertini Hospital, Rome, Italy; ^3^ Department of Pancreatic and Biliary Surgery, The First Affiliated Hospital of Harbin Medical University, Harbin, Heilongjiang, China; ^4^ Department of Gastroenterology, The First Affiliated Hospital, School of Medicine, Zhejiang University, Hangzhou, China; ^5^ Pancreatic Disease Centre, Department of Gastroenterology, The First Affiliated Hospital of Nanchang University, Nanchang, China; ^6^ Department of Surgery, University of Texas Health Sciences Center, Houston, TX, United States

**Keywords:** acute pancreatitis, damage associated molecular pattern, toll-like receptor, hypercholesterolemia, innate immune, adaptive immune

Acute pancreatitis is an common inflammatory gastrointestinal disease requiring acute admission to hospital (1). Most patients with acute pancreatitis have mild and self-limited symptoms, while up to 20% of patients develop severe disease requiring intensive care monitoring and support for organ failure (2).The incidence of AP is increasing at a rate of 2%-5% per year and varies between 3.4 and 73.4 cases per 100,000 worldwide (3). Despite the global burden of disease, there are currently no effective therapeutic agents to treat or prevent acute pancreatitis (4). Research into the pathogenesis of acute pancreatitis may help to identify novel cellular targets for potential drug development. There is increasing evidence that the severity and prognosis of acute pancreatitis are associated with uncontrolled or deregulated immune-system activation (4). This Research Topic highlights advances in the understanding of immune dysfunction in acute pancreatitis.

Normal gut mucosa is normally an effective barrier against the spread of toxins. Ischaemia and reperfusion of the intestinal mucosa during acute pancreatitis may damage the integrity of the intestinal barrier and increase intestinal permeability (5). The increased intestinal permeability could then lead to translocation of enteric bacteria and/or fungi into the pancreas, further contributing to the development of pancreatic necrosis and multiple organ failure. Reportedly, 68.8% of patients suffering from acute pancreatitis have bacterial DNA in their peripheral blood and over half (60.4%) display polymicrobial flora (6). Innate and adaptive immune cells stimulated by both pathogens and damage-associated molecular patterns (DAMPs) via pattern recognition receptors (PRRs) which including toll-like receptor-4 (TLR4) and dectin-1 (7), and the non-PRR DAMP receptors may be involved in amplifying proinflammatory cytokine responses and severe tissue injury (8). A mini-review by Otsuka et al. in this Research Topic summarises the role of fungus-induced activation of leucine-rich repeat kinase 2 (LRRK2) in the pathogenesis of severe acute pancreatitis. This study showed that the dectin-1-spleen tyrosine kinase (SYK)-leucine-rich repeat kinase 2 (LRRK2) axis, triggered by the detection of fungal cell wall elements, is capable of inducing the release of proinflammatory cytokines mediated by the nuclear translocation of NF-κB subunits.

TLR4 is present in a wide range of cells and has been shown to have several functions in the evolution of acute pancreatitis, independently of lipopolysaccharide (LPS) (9, 10). The initial insult of pancreatitis results in the release of intracellular contents from damaged cells into the extracellular space as DAMPs (such as HMGB1 and heat shock proteins), which act as key ligands for TLR4 and contribute to sterile inflammation (11). On the other hand, increased intestinal permeability and the entry of bacteria and/or bacterial products into the circulation could lead to increased exposure of acinar cells to gut bacterial components and activation of the NLRP3 (NOD-like receptor family pyrin domain containing-3) inflammasome through signalling via toll-like receptor 4 (TLR4) (10). Activation of the NLRP3 inflammasome leads to membrane pore formation and release of mature interleukin (IL)-1β and IL-18 from the cytoplasm, and enhances the systemic immune response by promoting neutrophil maturation, infiltration and macrophage infiltration (12). A review by Mattke et al. in this Research Topic summarises the relationship between TLR4 and pancreatic injury and immune infiltration in acute pancreatitis. They suggest that TLR4 plays a role not only in inflammation within acinar cells, but also with endothelial cells, macrophage maturation and polarization, and neutrophil activation and lifespan. And the activation of TLR4 leads not only to pancreatic damage but also to complications in other organs in acute pancreatitis.

Hypercholesterolemia, mainly caused by a high cholesterol diet, has been identified as a risk factor for severe acute pancreatitis (SAP) (13). Cholesterol accumulation favours the activation of both innate and adaptive inflammatory pathways, including the upregulation of TLR4 (14). Hypercholesterolaemia is capable of causing damage to endothelial cells through the production of nitric oxide and, in this way, leading to dysfunction of endothelial progenitor cells. Moreover, this endothelial microinflammation can lead to a reduction in blood supply, thus exacerbating the severity of the disease (14, 15). Despite these potential molecular mechanisms, the genomic relationship between a high cholesterol diet and AP has not been investigated. The study by Qiu et al. in this Research Topic identified the Fabp5 gene as the common differentially expressed gene between high cholesterol diet and acute pancreatitis. The Authors propose that by regulating the expression of Fabp5, resulting in increased activation of TLR 4 signalling and the nuclear factor-κB pathway, this type of nutrition may influence the severity of acute pancreatitis.

It is thought that neutrophils are the first immune cells to be recruited to inflammatory tissues in the setting of acute inflammation (4). Their immune functions are phagocytic, ROS production, degranulation and neutrophil extracellular trap (NET) formation and release (16, 17)]. Although NETs have a protective role in mediating host defence by trapping and killing microorganisms, the contents of NETs (histones and cell-free DNA) can be a source of DAMPs during sterile inflammation, which can be recognised by pattern recognition receptors, leading to the release of proinflammatory cytokines and ROS, and increased vascular permeability (12, 18). It is well known that NETs could be involved in the pathogenesis of AP by inducing trypsin activation, tissue damage and accelerating systemic inflammatory responses (10, 12). However, little is known about the mechanisms that control NET formation in AP. The study by Xu et al. in this Research Topic suggests that high expression of P-selectin may induce neutrophil extracellular traps via the PSGL-1/Syk/Ca2+/PAD4 pathway to exacerbate acute pancreatitis.

Immunoglobulin G (IgG) is the most abundant antibody in plasma and is a key molecule of the humoral immune response, linking innate and adaptive immunity through its multiple roles (19). N-glycans attach to the conserved asparagine 297 in the fragment crystallizable (Fc) portion of this molecule (altering IgG conformation and interactions with various receptors) and act as a switch between pro- and anti-inflammatory IgG functionality (20). IgG N-glycosylation affects physiology and an altered profile of IgG N-glycans contributes to disease progression and has been implicated in autoimmune pancreatitis, type 2 diabetes and cardiovascular disease (21, 22). The study by Chen et al. in this Research Topic investigated the causality of genetically predicted IgG N-glycosylation traits on different forms of pancreatitis using Mendelian Randomisation (MR) analysis, which may be helpful in understanding the role of immune cells and the microenvironment in the pathogenesis of pancreatitis.

Evidence suggests that severe acute pancreatitis shares many clinical and biochemical features with sepsis syndrome and septic shock (23). In both severe acute pancreatitis and sepsis, the major cytokines involved in the development of complications are tumour necrosis factor-alpha, interleukin-1, interleukin-6 and interleukin-8 (23). Microbial translocation as a result of altered gut permeability may be a major cause of sepsis-related morbidity in patients presenting with AP (24).Therefore, it is hypothesised that acute pancreatitis and sepsis may share a common pathogenesis. The study by Liu et al. in this Research Topic investigated the causal relationship between acute pancreatitis and sepsis, as well as the causal association of 731 immune cell characteristics with acute pancreatitis and sepsis by Mendelian randomisation (MR) analysis. They indicated that memory CD8+ T cells may play a crucial role in the evolution from AP to sepsis.

Taken together, both innate and adaptive immune responses are implicated in the mechanisms underlying pancreatitis (25, 26). Despite the aforementioned advances in knowing the pathogenesis, there are currently no effective immunotherapeutic agents for the treatment of acute pancreatitis (4). Despite improvements in treatment and critical care, severe acute pancreatitis is still associated with high mortality (1), and there are currently no specific pharmacological therapies with proven efficacy (27). Therefore, further studies that elucidate the role of immune dysfunction during disease progression may open new avenues for immunomodulatory therapy in acute pancreatitis.

## Author contributions

WDH: Conceptualization, Data curation, Formal analysis, Funding acquisition, Investigation, Methodology, Project administration, Resources, Software, Supervision, Validation, Visualization, Writing – original draft, Writing – review & editing. MZ: Writing – original draft, Writing – review & editing. GW: Writing – review & editing. XJ: Writing – review & editing. WHH: Writing – review & editing. HG: Writing – review & editing.
